# The search for an HIV vaccine, the journey continues

**DOI:** 10.1002/jia2.25506

**Published:** 2020-05-16

**Authors:** Carl W Dieffenbach, Anthony S Fauci

**Affiliations:** ^1^ Division of AIDS National Institute of Allergy and Infectious Diseases National Institutes of Health Bethesda MD USA; ^2^ Office of the Director National Institute of Allergy and Infectious Diseases National Institutes of Health Bethesda MD USA

**Keywords:** HIV Vaccine, correlates of protection, clinical trials, broad neutralizing antibody, clade, immunogen design

As we commemorate HIV Vaccine Awareness Day, we honour the thousands of study participants and the research teams that have played essential roles in the completed and ongoing vaccine clinical trials. We also recognize the scientists who have spent years dedicated to creating the tools and technologies to end the global HIV epidemic. On this Vaccine Awareness Day, we must acknowledge that the HIV vaccine research field is quickly approaching a critical crossroads. This past decade has been spent launching improved vaccine concepts and immune‐based strategies into trials. Despite these advances, a safe and durably effective HIV vaccine has eluded us. In this regard, we must evaluate the current state of the field and make appropriate adjustments to help speed us to our goal. While this quest for a vaccine at times may feel extremely difficult, as evidenced by the recent disappointing results of a phase III efficacy trial, HVTN 702, described below, we must steadfastly move forward to address critical research gaps and unanswered questions.

As we reflect upon the status of HIV vaccine research, we would be remiss if we did not mention the current global pandemic caused by SARS‐CoV‐2. It is important to acknowledge the global response to the COVID‐19 epidemic and to cite the range of vaccine platforms being used for anti‐SARS‐CoV‐2 candidate vaccines that have their origin in HIV vaccinology. The HIV vaccine field is clearly assisting in addressing this newly emergent pandemic and the National Institute of Allergy and Infectious Diseases (NIAID) is fostering these essential collaborations. For HIV Vaccine Awareness Day in 2020, we emphatically state that finding safe, effective and durable vaccines for HIV and COVID‐19 are NIAID’s top priorities. The world must have both.

Based upon the challenges that HIV biology presents: global sequence diversity, integration of the viral genome into the host cells and long duration of the symptom free period of infection as well as the fact that no single individual has been spontaneously cured, it is widely accepted that a vaccine must trigger responses that are qualitatively different from the immune responses to natural infection. After the STEP trial (also referred to as HVTN 502 or Merck V520‐023) was halted in 2007, vaccine candidates that fulfilled these criteria have been prioritized [1, 2]. Over the past decade, three different vaccine approaches have been implemented, possible correlates of protection identified, and two have moved through clinical evaluation to advanced clinical trials.

The first of these was based upon the correlates of risk that were generated from analysis of the RV144 trial conducted in Thailand, which in 2009, reported modest levels of efficacy [3, 4]. Rather than perform a direct repeat of RV144 in Thailand, where the epidemic is driven by viruses from clades AE and B, a clade C‐specific regimen was developed for testing in Southern Africa. When evaluated in the HVTN100 trial, the relevant correlate responses were detected and shown to be longer lasting than those of RV144 [5, 6, 7]. With the go‐no‐go criteria for advancement met, the HVTN702 study moved ahead. However, no protection was observed, and the trial was terminated due to futility. While the field is disappointed with this outcome, it is important to understand the possible reasons for why this occurred. First, there were significant differences in the overall incidence between the study cohorts in HVTN702 and RV144 that could have overwhelmed a modestly protective response. Additionally, it is conceivable that the correlates observed in RV144 are specific to the clades and population in Thailand and not transferable to the Southern Africa epidemic.

The remaining two vaccine concepts have reproducibly demonstrated efficacy in non‐human primate studies. The most advanced concept is currently being evaluated in two studies, Imbokodo (HVTN705/HPX2008) in women in Southern Africa and Mosaico (HVTN706/HPX3002) in men and transgender persons who have sex with men in the Americas and Europe [8]. Analysis of the correlates of protection seen in the non‐human primate studies point to qualitatively different responses than those observed in RV144, and the trials are evaluating *in silico* designed immunogens to present the most globally conserved HIV sequences to trigger quantitatively superior CD8 + T cell responses [8, 9]. The third approach, still in preclinical studies, is an SIV vaccine delivered via the rhesus cytomegalovirus (RhCMV) vector platform. This strategy has been shown by Picker and colleagues to protect 50 percent of monkeys from sustained infection [10]. Efforts are currently underway to transition to a human CMV vector platform with Phase 1 clinical trials expected in 2021.

Although humbled by the outcome of HVTN702, we must continue to explore novel methods of inducing or providing protective immunity against HIV infection. One of the most exciting developments in HIV research has been the ability to isolate and produce broadly neutralizing monoclonal antibodies (bNAbs) that neutralize nearly all of circulating virus strains from all clades [11, 12]. These human antibodies prevent acquisition of SHIV infection, in a dose dependent manner, in animal challenge models and when combinations of these antibodies are used in people living with HIV, strong antiviral activity has been demonstrated. The Antibody Mediated Protection (AMP) trials are currently evaluating VRC01, the CD4 binding site targeted bNAb, to determine the ability of this single antibody to prevent HIV infection in women in Southern Africa and MSM and transgender persons in the Americas [13]. The AMP studies have not been impacted by COVID‐19, and results from the AMP studies are expected in fall of 2020. If protection is observed in the AMP trials, an important correlate for future vaccine studies will be established.

In the meantime, efforts are underway to define immunogens that specifically trigger neutralizing antibodies by targeting five specific epitopes, which are unique sites of vulnerability on the HIV Env trimer glycoprotein [12, 14]. The goal is to create a discrete set of immunogens that can reproducibly elicit the production of neutralizing antibody responses against two or more of these target sites. The Collaborative HIV Immunogen Project [12] is seeking to prospectively design and select the optimal combination of immunogens to be evaluated for the induction of broad neutralization of a range of HIV isolates.

As we reflect on HIV Vaccine Awareness Day and recent developments in the vaccine field, it is clear that much work remains to be done. Going forward we must continue to engage community to ensure a shared understanding of our common goals. While we pursue a safe and effective HIV vaccine, we are also seeking ways of improving chemoprophylaxis. We remain optimistic that effective interventions that people will reliably use can be developed and brought to scale. On HIV Vaccine Awareness Day, let us pause and reflect on how far we have come while continuing to focus on completing the work needed to achieve an effective vaccine.

## COMPETING INTERESTS

ASF and CWD have no competing financial interests.

## AUTHORS’ CONTRIBUTIONS

The authors collaboratively conceived the content of the paper. CWD wrote the first draft and ASF edited the manuscript into this final form.
